# Epidemiology of congenital diaphragmatic hernia among 24 million Chinese births: a hospital-based surveillance study

**DOI:** 10.1007/s12519-023-00774-y

**Published:** 2023-12-09

**Authors:** Zhi-Yu Chen, Jing Tao, Wen-Li Xu, Yu-Yang Gao, Wen-Yan Li, Zhen Liu, Jia-Yuan Zhou, Li Dai

**Affiliations:** 1 National Center for Birth Defects Monitoring, West China Second University Hospital, Sichuan University, No. 17 Section 3 Renminnanlu, Chengdu 610041, China; 2 The Joint Laboratory for Pulmonary Development and Related Diseases, West China Institute of Women and Children’s Health, West China Second University Hospital, Sichuan University, Chengdu, China; 3 Key Laboratory of Birth Defects and Related Diseases of Women and Children, Ministry of Education, Sichuan University, Chengdu, China; 4 NHC Key Laboratory of Chronobology, Sichuan University, Chengdu, China; 5 Med-X Center for Informatics, Sichuan University, Chengdu, China

**Keywords:** China, Congenital diaphragmatic hernia, Epidemiology, Prevalence

## Abstract

**Background:**

The prevalence of congenital diaphragmatic hernia (CDH) varies across countries, with limited information available on its epidemiology in China. Our study aimed to investigate the prevalence, time trends, and perinatal outcomes of CDH in China, as well as its associated malformations and potential associations with maternal and infant characteristics.

**Methods:**

This study included all birth and CDH cases from the Chinese Birth Defects Monitoring Network between 2007 and 2019, with CDH cases classified as either isolated or associated. We employed the joinpoint regression model to calculate the trends of prevalence and the annual percent change, with Poisson regression used for adjusted prevalence rate ratios. A *P* value ≤ 0.05 was considered statistically significant.

**Results:**

A total of 4397 CDH cases were identified among 24,158,029 births in the study period, yielding prevalence rates of 1.82, 1.13 and 0.69 per 10,000 for overall, isolated, and associated CDH, respectively. The prevalence of each type of CDH increased over time. The prevalence of overall CDH varied significantly by infant sex (male vs. female, 1.91/10,000 vs. 1.63/10,000), maternal residence (urban vs. rural, 2.13/10,000 vs. 1.45/10,000), maternal age (< 20 years, 1.31/10,000; 20–24 years, 1.63/10,000; 25–29 years, 1.80/10,000; 30–34 years, 1.87/10,000; ≥ 35 years, 2.22/10,000), and geographic region (central, 1.64/10,000; east, 2.45/10,000; west, 1.37/10,000). Cardiovascular anomalies were the most common malformations associated with CDH. Infants with associated CDH had a higher risk of premature birth and perinatal death than those with isolated CDH.

**Conclusion:**

The increasing prevalence and high perinatal mortality rate of CDH highlight the need for further etiological, epidemiological, and clinical studies among the Chinese population.

**Video Abstract**

**Supplementary Information:**

The online version contains supplementary material available at 10.1007/s12519-023-00774-y.

## Introduction

Congenital diaphragmatic hernia (CDH) is a structural birth defect characterized by incomplete formation of the diaphragm, which allows abdominal organs to herniate into the thoracic cavity and impair pulmonary and vascular development. The diaphragm typically develops between the 4th and 8th weeks of gestation [1]. Most cases of CDH are left-sided but right-sided, and in rare cases, bilateral hernias can also occur [2]. The vast majority of CDH cases are isolated, with only approximately one-third being associated with other anomalies [3].

Previous studies have reported that the prevalence rate of CDH in the Chinese population was 0.7 per 10,000 births during the period from 1986 to 1993 [4]. In contrast, the prevalence of CDH in other countries has been reported to range from 1.93 to 3.80 per 10,000 births, depending on the study population and time period [1, 5–9]. Regardless of the specific type of CDH, all cases are associated with a poor prognosis. The overall perinatal mortality rate for CDH has been reported to be as high as 84.1% in China [4], 65.5% in Australia [9] and 32.7% for stillbirths and early neonatal death in the United States [7]. Although the pathogenesis of CDH remains largely unknown, several potential teratogenic causes have been proposed, including quinine, phenmetrazine, nitrofen, and vitamin A deficiency [10–12]. In addition, maternal age, maternal residence, and male birth have also been suggested as factors associated with the prevalence of CDH, but the correlations are inconsistent [1, 6, 7, 13, 14]. In recent decades, China has undergone significant changes in its economy, environment, and maternal and child health [15]. To gain new insights into the epidemiology of CDH, we conducted a study using data from the Chinese Birth Defects Monitoring Network (CBDMN) from 2007 to 2019. Our study aimed to investigate the prevalence and time trends of CDH, with a particular focus on perinatal outcomes, associated malformations, and potential associations with maternal and infant characteristics.

## Methods

### Data source and quality

The CBDMN is a well-established, nationwide hospital-based birth defect surveillance system with the largest sample size and geographical coverage in China, managed by the National Health Commission. It covers 763 member hospitals in 31 provinces, municipalities, or autonomous regions and currently monitors approximately 1.6 million births annually, accounting for more than 10% of annual births in China [16]. For this study, data from 1 January 2007 to 31 December 2019 were derived from the CBDMN. All births (live birth, stillbirths, and terminations of pregnancy) with a gestational age ≥ 28 weeks, obtained from delivery or medical records in member hospitals, were examined for congenital malformations by trained obstetrics, pediatrics, and ultrasound experts. The maximum diagnosis time for a congenital malformation case was the seventh day after birth. At each member hospital, trained professionals collected data on births and birth defect cases using standardized forms. All anomalies in the CBDMN database were coded according to the International Classification of Disease 10th version (ICD-10) by a national panel. A detailed description of the three-level (county, provincial, and national) data collection and quality management network can be found elsewhere [17, 18]. Quality control of the data was performed routinely through an independent retrospective survey. A panel of senior health professionals at the three levels evaluated the completeness, accuracy, and timeliness of the data. At the county level, all data reported by member hospitals were investigated quarterly. At the provincial and national levels, data reported by approximately one-third of the member hospitals were investigated semiannually and annually, respectively. More details about the CBDMN have been published elsewhere [16, 18–20]. Ethical approval was not necessary since the study was based on anonymized routine surveillance data with no identifiable information on mothers. Permission was obtained from the National Health Commission of China to use the data for this analysis.

### Case identification and classification

The CBDMN defined CDH as a congenital malformation characterized by herniation of abdominal contents into the thorax through a diaphragmatic defect, including total absence of the diaphragm but excluding diaphragmatic paralysis, diaphragmatic eventration, or hiatal hernia [13, 21]. CDH corresponds to ICD-10 code “Q79.0”. Diagnostic approaches commonly used to identify CDH cases include ultrasound sonography, magnetic resonance imaging, and X rays. In our study, CDH cases were classified into two mutually exclusive groups: isolated, defined as the absence of any other Q or ICD-10 codes other than Q79.0 in the CBDMN register, and associated or non-isolated, when other additional codes existed.

### Statistical standards and methods

The prevalence rate of CDH was calculated as the number of cases of CDH per 10,000 births (live births, stillbirths and terminations of greater than or equal to 28 weeks of gestational age). The rates were analyzed according to the following demographic characteristics: year of birth, geographic location, maternal residence, maternal age, and infant sex. Maternal residence was defined as the mothers’ residence during pregnancy and was classified as urban (cities and urbanized areas/towns) or rural (villages or countryside) based on the mother’s last residence for at least one year [17]. In our study, we classified China’s 31 provinces into three regions based on their geographical location and level of economic development [18]. The central region included 10 provinces: Hebei, Shanxi, Jilin, Heilongjiang, Anhui, Jiangxi, Henan, Hubei, Hunan, and Hainan. The eastern region included nine provinces: Beijing, Tianjin, Liaoning, Shanghai, Jiangsu, Zhejiang, Fujian, Shandong, and Guangzhou. The western region included 12 provinces: Inner Mongolia, Guangxi, Sichuan, Chongqing, Guizhou, Yunnan, Xizang, Shaanxi, Gansu, Qinghai, Ningxia and Xinjiang. Maternal age was categorized into five age groups: < 20 years, 20–24 years, 25–29 years, 30–34 years, and ≥ 35 years [20]. We also compared the timing of diagnosis (postpartum and antenatal), distribution of gestational age (< 37, 37–42, and ≥ 42 weeks of gestation), and perinatal outcome (stillbirths/terminations, early neonate death and alive within 7 days) between isolated and associated cases. Additionally, we analyzed the differences in demographic and associated deformity characteristics between CDH cases that survived and those that did not.

Prevalence rates and their 95% confidence intervals (95% CI) were estimated using the Poisson distribution. Poisson regression was used to calculate the adjusted prevalence rate ratios (aPRRs) and their 95% CIs. When calculating the aPRR for each factor (birth year, maternal residence, maternal age, geographic region, and infant sex), we controlled for the effects of others. Time trends in prevalence over the study period were analyzed using joinpoint regression. Changes in the prevalence of overall, isolated and associated CDH are presented as the average annual percentage change. Chi-square tests were used to examine differences in the timing of diagnosis and perinatal outcomes between isolated and associated cases, as well as differences in demographic characteristics and associated deformities between surviving and deceased cases. Data analysis was performed using R version 4.0.2 (the Comprehensive R Archive Network: http://cran.r-project.org). Additionally, Joinpoint regression was conducted using the Joinpoint Regression Program (version 4.9.0.1; Statistical Research and Applications Branch, National Cancer Institute, Bethesda, MD, USA). The statistical significance level for *α* was set at 0.05.

## Results

### Prevalence rates of CDH

Between 2007 and 2019, we identified a total of 4397 cases of CDH among 24,158,029 births. Of these cases, 2737 (62.2%) were isolated, and 1660 (37.8%) were associated with other conditions. The prevalence rate of overall CDH was 1.82 per 10,000 births (95% CI = 1.77–1.87), with a prevalence of 1.13 per 10,000 births (95% CI = 1.09–1.18) for isolated CDH and 0.69 per 10,000 births (95% CI = 0.65–0.72) for associated CDH.

**Fig. 1:**
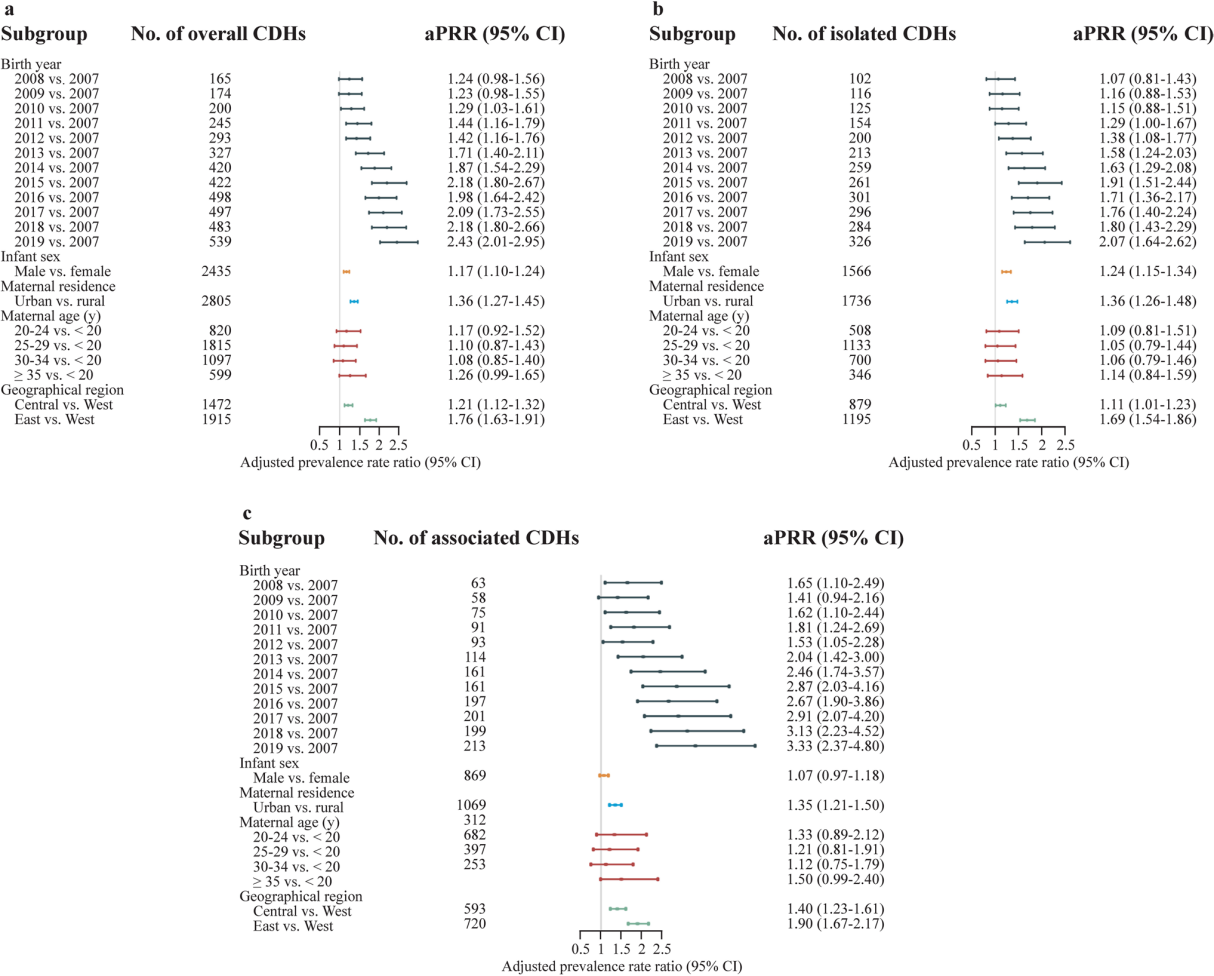
The adjusted prevalence rate ratio (aPRR) and 95% confidence intervals (CIs) of congenital diaphragmatic hernia (CDH). **a** The aPRR and 95% CIs of overall CDHs; **b** the aPRR and 95% CIs of isolated CDHs; **c** the aPRR and 95% CIs of associated CDHs

Table 1 displays the prevalence of CDH by selected demographic characteristics. The prevalence of both overall and isolated CDH showed a considerable male predominance. Mothers residing in urban areas had a significantly higher probability of having infants with CDH than those residing in rural areas (Table 1, Fig. 1). An upward trend was observed for maternal age-specific prevalence rates of overall, isolated, and associated CDH (Table 1). However, after accounting for heterogeneity between maternal residence, geographic region, infant sex and year of delivery, no statistically significant associations were found with maternal age (Fig. 1). Compared to newborns whose mothers resided in western regions, newborns whose mothers resided in central regions had a 1.10–1.37-fold higher prevalence rate of overall, isolated and associated CDH, while newborns whose mothers resided in eastern regions had a 1.72–1.89-fold higher prevalence rate of these conditions (Table 1, Fig. 1).

**Table 1:** Prevalence rates of congenital diaphragmatic hernia stratified by birth year, infant sex, maternal residence, maternal age and geographic region (per 10,000 births)

Characteristics	Number of births	Overall		Isolated		Associated	
		Cases	Prevalence (95% CI)	Cases	Prevalence (95% CI)	Cases	Prevalence (95% CI)
Birth year							
2007	1,258,298	134	1.06 (0.89–1.26)	95	0.75 (0.61–0.92)	39	0.31 (0.22–0.42)
2008	1,314,091	165	1.26 (1.07–1.46)	102	0.78 (0.63–0.94)	63	0.48 (0.37–0.61)
2009	1,401,331	174	1.24 (1.06–1.44)	116	0.83 (0.68–0.99)	58	0.41 (0.31–0.54)
2010	1,531,143	200	1.31 (1.13–1.50)	126	0.82 (0.68–0.97)	74	0.48 (0.38–0.61)
2011	1,681,096	245	1.46 (1.28–1.65)	154	0.92 (0.78–1.07)	91	0.54 (0.44–0.66)
2012	2,005,526	293	1.46 (1.30–1.64)	200	1.00 (0.86–1.15)	93	0.46 (0.37–0.57)
2013	1,893,854	327	1.73 (1.54–1.92)	213	1.12 (0.98–1.29)	114	0.60 (0.50–0.72)
2014	2,198,818	420	1.91 (1.73–2.10)	260	1.18 (1.04–1.34)	160	0.73 (0.62–0.85)
2015	1,883,843	422	2.24 (2.03–2.46)	261	1.39 (1.22–1.56)	161	0.85 (0.73–1.00)
2016	2,432,979	498	2.05 (1.87–2.23)	302	1.24 (1.10–1.39)	196	0.81 (0.70–0.93)
2017	2,315,621	497	2.15 (1.96–2.34)	296	1.28 (1.14–1.43)	201	0.87 (0.75–1.00)
2018	2,097,800	483	2.30 (2.10–2.52)	285	1.36 (1.21–1.53)	198	0.94 (0.82–1.08)
2019	2,143,629	539	2.51 (2.31–2.74)	327	1.53 (1.36–1.70)	212	0.99 (0.86–1.13)
Infant sex^a^							
Male	12,774,247	2435	1.91 (1.83–1.98)	1570	1.23 (1.17–1.29)	865	0.68 (0.63–0.72)
Female	11,379,102	1859	1.63 (0.89–1.26)	1132	0.99 (0.94–1.05)	727	0.64 (0.59–0.69)
Maternal residence							
Urban	13,184,097	2805	2.13 (2.05–2.21)	1740	1.32 (1.26–1.38)	1065	0.81 (0.76–0.86)
Rural	10,973,932	1592	1.45 (1.38–1.52)	997	0.91 (0.85–0.97)	595	0.54 (0.50–0.59)
Maternal age (y)							
< 20	503,203	66	1.31 (1.01–1.67)	44	0.87 (0.64–1.17)	22	0.44 (0.27–0.66)
20–24	5,015,677	820	1.63 (1.52–1.75)	509	1.01 (0.93–1.11)	311	0.62 (0.55–0.69)
25–29	10,074,584	1815	1.80 (1.72–1.89)	1135	1.13 (1.06–1.19)	680	0.67 (0.63–0.73)
30–34	5,864,833	1097	1.87 (1.76–1.98)	702	1.20 (1.11–1.29)	395	0.67 (0.61–0.74)
≥ 35	2,699,732	599	2.22 (2.04–2.40)	347	1.29 (1.15–1.43)	252	0.93 (0.82–1.06)
Geographic region							
Central	8,966,230	1472	1.64 (1.56–1.73)	880	0.98 (0.92–1.05)	592	0.66 (0.61–0.72)
East	7,820,462	1915	2.45 (2.34–2.56)	1200	1.53 (1.45–1.62)	715	0.91 (0.85–0.98)
West	7,371,337	1010	1.37 (1.29–1.46)	657	0.89 (0.82–0.96)	353	0.48 (0.43–0.53)

*CI* confidence interval. ^a^One hundred and three cases and 4680 perinatal infants with unknown/unspecified gender were excluded

### The trend of congenital diaphragmatic hernia

**Fig. 2:**
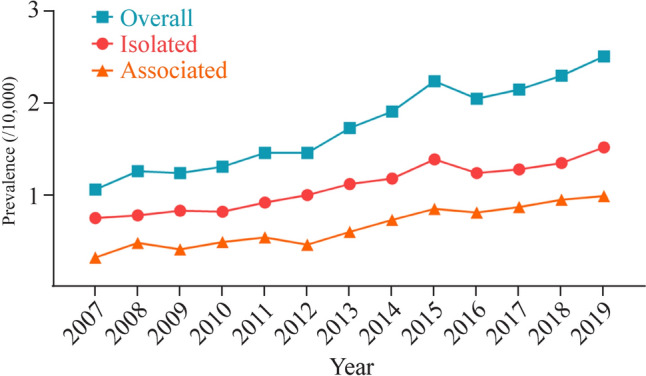
Time trends in the prevalence of congenital diaphragmatic hernia in Chinese newborns, 2007–2019. Overall: annual percentage change (APC) = 7.32, *P* < 0.001; isolated: APC = 6.24, *P* < 0.001; associated: APC = 9.35, *P* < 0.001

From 2007 to 2019, the annual prevalence rates of overall, isolated, and associated CDH increased from 1.06/10,000 to 2.51/10,000, from 0.75/10,000 to 1.53/10,000, and from 0.31/10,000 to 0.99/10,000, respectively (Table 1 and Fig. 2). The prevalence rate of associated CDH showed the highest upward trend, with an annual percent change of 9.4%, followed by a significant increase in the overall prevalence rate of 7.3%. In comparison, the prevalence of isolated cases rose moderately by 6.2% per year. Compared to 2007, the prevalence of isolated CDH in 2019 increased by one time, while the prevalence of associated CDH increased by three times.

### Prenatal detection

As shown in Supplementary Table 1, antenatal diagnosis was available for 87.4% of the overall cases. Among the 3843 patients, 2669 (69.5%) underwent termination of pregnancy. The median gestational age at prenatal diagnosis for overall, isolated and associated cases was 25, 26 and 25 weeks, respectively.

### Associated anomalies

Of the 1660 CDH cases with additional anomalies, 49.5% of associated cases had only one additional malformation, while 50.5% had two or more extra anomalies. Cardiovascular anomalies were the most commonly associated with CDH, occurring in 999 (60.2%) cases, followed by musculoskeletal anomalies (18.7%). Chromosomal abnormalities were present in 51 (3.1%) cases (Table 2).

**Table 2:** Abnormalities associated with congenital diaphragmatic hernia

System/abnormalities	ICD-10 code	Cases^a^	Percent
Nervous system	Q00–Q07	284	17.11
Anencephaly	Q00	25	1.51
Encephalocele	Q01	20	1.20
Microcephaly	Q02	4	0.24
Hydrocephalus	Q03	108	6.51
Other malformations of brain	Q04	109	6.57
Spina bifida	Q05	45	2.71
Other congenital malformations of spinal cord	Q06	1	0.06
Other malformations of nervous system	Q07	1	0.06
Eye, ear, face and neck	Q10–Q18	60	3.61
Congenital malformations of eyelid, lacrimal apparatus, and orbit	Q10	1	0.06
Anophthalmos, microphthalmos, and macrophthalmos	Q11	8	0.48
Congenital lens malformations	Q12	1	0.06
Congenital malformations of posterior segment of eye	Q14	2	0.12
Other congenital malformations of eye	Q15	7	0.42
Congenital malformations of ear causing impairment of hearing	Q16	7	0.42
Malformations of ear	Q17	28	1.69
Malformations of face and neck	Q18	9	0.54
Circulatory system	Q20–Q28	999	60.18
Malformations of cardiac chambers and connections	Q20	128	7.71
Malformations of cardiac septa	Q21	514	30.96
Congenital malformations of pulmonary and tricuspid valves	Q22	54	3.25
Congenital malformations of aortic and mitral valves	Q23	43	2.59
Other congenital malformations of heart	Q24	352	21.20
Congenital malformations of great arteries	Q25	252	15.18
Congenital malformations of great veins	Q26	43	2.59
Other congenital malformations of peripheral vascular system	Q27	116	6.99
Respiratory system	Q30–Q34	199	11.99
Congenital malformations of nose	Q30	32	1.93
Congenital malformations of lung	Q33	168	10.12
Cleft lip and cleft palate	Q35–Q37	136	8.19
Cleft palate	Q35	21	1.27
Cleft lip	Q36	31	1.87
Cleft palate with cleft lip	Q37	84	5.06
Digestive system	Q38–Q45	94	5.66
Other congenital malformations of tongue, mouth and pharynx	Q38	2	0.12
Congenital malformations of esophagus	Q39	11	0.66
Other congenital malformations of upper alimentary tract	Q40	14	0.84
Congenital absence, atresia and stenosis of small intestine	Q41	14	0.84
Congenital absence, atresia and stenosis of large intestine	Q42	13	0.78
Other congenital malformations of intestine	Q43	21	1.27
Congenital malformations of gallbladder, bile ducts, and liver	Q44	20	1.20
Other congenital malformations of digestive system	Q45	6	0.36
Genital organs	Q50–Q56	50	3.01
Congenital malformations of uterus and cervix	Q51	1	0.06
Other congenital malformations of female genitalia	Q52	1	0.06
Undescended testicle	Q53	21	1.27
Hypospadias	Q54	10	0.60
Other congenital malformations of male genital organs	Q55	7	0.42
Indeterminate sex and pseudohermaphroditism	Q56	11	0.66
Urinary system	Q60–Q64	163	9.82
Renal agenesis and other reduction defects of kidney	Q60	30	1.81
Cystic kidney disease	Q61	41	2.47
Malformations of renal pelvis and ureter	Q62	36	2.17
Other malformations of kidney	Q63	60	3.61
Other congenital malformations of urinary system	Q64	4	0.24
Musculoskeletal system	Q65–Q79	311	18.73
Congenital deformities of hip	Q65	1	0.06
Congenital deformities of feet	Q66	43	2.59
Congenital musculoskeletal deformities of head, face, spine, and chest	Q67	10	0.60
Other congenital musculoskeletal deformities	Q68	13	0.78
Polydactyly	Q69	38	2.29
Syndactyly	Q70	24	1.45
Reduction defects of upper limb	Q71	54	3.25
Reduction defects of lower limb	Q72	20	1.20
Reduction defects of unspecified limb	Q73	7	0.42
Other congenital malformations of limb(s)	Q74	22	1.33
Other congenital malformations of skull and face bones	Q75	12	0.72
Other congenital malformations of spine and bony thorax	Q76	49	2.95
Osteochondrodysplasia with defects of growth of tubular bones and spine	Q77	1	0.06
Other malformations of musculoskeletal system	Q79	78	4.70
Chromosomal abnormalities	Q90–Q99	51	3.07
Down’s syndrome	Q90	7	0.42
Edward’s syndrome	Q91.3	14	0.84
Patau’s syndrome	Q91.7	2	0.12
Monosomies and deletions from the autosomes, not elsewhere classified	Q93	4	0.24
Other sex chromosome abnormalities, female phenotype, not elsewhere classified	Q97	1	0.06
Other chromosome abnormalities, not elsewhere classified	Q99	24	1.45
Other malformations	Q80–Q89	55	3.31
Other congenital malformations of skin	Q82	1	0.06
Other specified congenital malformation syndromes affecting multiple systems	Q87	8	0.48
Other congenital malformations, not elsewhere classified	Q89	48	2.89
Other malformations, not coded in Q00–Q99	–	106	6.39

^a^The number of cases were counted by patients

### Perinatal outcomes

Table 3 shows the perinatal outcomes of CDH cases. Preterm births accounted for 66.2% of overall cases, with more associated CDHs born prematurely than isolated cases. Only 29.8% of CDH cases survived the perinatal period, with a lower survival rate among associated cases (19.9%). The perinatal mortality rates were 70.3% for overall cases, 80.1% for associated CDH, and 64.2% for isolated cases.

**Table 3:** Perinatal outcomes of congenital diaphragmatic hernia cases in Chinese newborns

Perinatal outcomes	Overall (*N* = 4397)	Isolated (*n* = 2737)	Associated (*n* = 1660)
Gestational age (wk)^a,*^			
< 37	2907 (66.2)	1642 (60.1)	1265 (76.3)
37–42	1463 (33.3)	1073 (39.2)	390 (23.5)
≥ 42	22 (0.5)	19 (0.7)	3 (0.2)
Perinatal outcome^b,*^			
Stillbirths/terminations	2725 (62.2)	1509 (55.3)	1216 (73.5)
Early neonate death	353 (8.1)	244 (8.9)	109 (6.6)
Alive within 7 d	1306 (29.8)	977 (35.8)	329 (19.9)

Data are presented as *n* (%). ^a^Include all the live births, stillbirths and terminations excluded five cases with unknown gestational age; ^b^include all the live births, stillbirths and terminations excluded 13 cases with unknown perinatal outcome. ^*^Differed significantly between isolated and associated

Among surviving cases, a higher proportion were male infants or from the eastern geographic regions. Among deceased cases, almost 95.0% were diagnosed prenatally, and 86.6% were of low gestational age (Supplementary Table 2). The percentage of concomitant cardiovascular system abnormalities was higher among surviving cases, while nervous system and musculoskeletal system abnormalities were more common among deceased cases (Supplementary Table 3).

## Discussion

Our study found that the prevalence of CDH was 1.82 per 10,000 births and showed an increasing trend over time. Higher prevalence rates were observed among male fetuses, older mothers, and mothers residing in urban areas, particularly in the eastern and central regions. Additionally, infants with associated CDH had a higher risk of premature birth and perinatal death compared to those with isolated CDH.

The prevalence rate of overall CDH is lower than rates reported in studies from the United States [7], France [22], Australia [9], Finland [23], Croatia [24], Sweden [25], and other European countries [1]. One possible explanation for the difference between our study and others is that our surveillance is hospital-based, while others are population-based. However, when compared to other hospital-based surveillance programs in Argentina, Cuba, Japan, and Saudi Arabia, our prevalence is still lower [21, 26]. Our study also revealed a lower prevalence in both isolated (1.13/10,000 births) and associated cases (0.69/10,000 births) [1, 6]. Factors such as birth definition, racial characteristics, and time range may contribute to these differences in prevalence rates.

An increasing trend in the prevalence of CDH was observed during the study period. However, trends in CDH prevalence varied across different countries and regions. For example, no change was seen in California from 1989 to 1997 [6] or in the United States from 1995 to 2002 [7]. In contrast, our analysis showed an upward trend in CDH prevalence over time, consistent with the studies conducted in the Middle East, Europe, North America, Central America, and South America [1, 21]. Our upward trend was even more significant, with an average annual percent change of 7.32%, compared to 0.47% in the aforementioned regions [21]. The rising trend of CDH prevalence in China may be attributed to improvements in prenatal diagnosis, including better and more accessible ultrasonography. Changes in maternal environmental exposures and other factors associated with CDH may also contribute to the increasing trend.

Our study showed a higher risk of CDH among older mothers, consistent with previous studies [6, 7, 27, 28], which was also comparable to studies that found no association or observed a slight, non-significant increase in prevalence among older maternal age groups [1, 6, 29]. Furthermore, our finding that males are at an approximately 20% higher risk than females for developing CDH is in general agreement with previous studies [6, 7, 13, 30]. In our study, higher prevalence rates were found in the eastern and central regions and in urban areas, suggesting a possible role for environmental factors in the pathogenesis of CDH.

CDH can be diagnosed prenatally or postnatally. The percentage of patients with CDHs diagnosed prenatally by ultrasound has significantly increased over the last 20 years, from 15% to 50%–75% [31]. In our study, 87.4% of the overall CDH cases were diagnosed prenatally, close to the 84.1% reported in other findings [32]. This is likely due to advances in ultrasonic diagnosis technology. Consistent with previous studies [1, 22, 33, 34], we found that the prenatal detection rate was higher for the non-isolated CDH cases than for the isolated cases. This may be because non-isolated CDH can be detected earlier by prenatal ultrasound [22, 33]. Our study and others confirm that there is large national variation in the prenatal detection rate of CDH due to differences in policies regarding antenatal routine ultrasound screening [1]. Associated anomalies may also contribute to the prenatal detection of CDH fetuses.

CDH is known to be associated with other structural anomalies and chromosome abnormalities, with the proportion of associated anomalies ranging from 28.2% to 85.3% [1, 6, 9, 13]. The large range of changes may be due to the differences in the CDH case confirmation and data sources. In our analysis, approximately one-third of CDH patients had associated anomalies, consistent with other studies [3, 13, 21, 31, 35]. Specifically, we observed the highest frequency of co-occurring cardiovascular anomalies, followed by musculoskeletal anomalies and nervous system malformations, which are the same as those of previous investigations [6]. Accompanying the high incidence of congenital heart disease in CDH is increasing evidence of fetal ventricular hypoplasia, characterized by a narrowing and elongation of the left ventricle [36–39]. The development of fetal left ventricle hypoplasia is likely multifactorial, secondary to direct mechanical compression of the left ventricle by the herniated abdominal viscera and flow-related mechanisms [40]. Left ventricle hypoplasia could be a risk factor for early postnatal ventricular dysfunction, increasingly recognized as a contributor to CDH pathophysiology and outcome [41].

Mortality rates for CDH patients vary considerably in the literature. Our study found an overall perinatal mortality rate of 70.3%, higher than the mortality of 42%–68% reported in other studies [9, 42, 43]. Differences in measuring mortality among CDH patients make it difficult to accurately evaluate variations between studies, which may be due to the presence of “hidden mortality” [42, 44]. “Hidden mortality” refers to the exclusion of intrauterine deaths and induced terminations from institution-based studies. Therefore, recently reported increases in survival rates should be interpreted with caution. Our study found that the mortality rate of stillbirths and terminations was 62.2%, which partially accounted for the “hidden mortality”. Despite accounting for “hidden deaths” in our mortality calculations, our study still reported a higher perinatal mortality rate compared to the literature [22, 42, 45]. This variation may be attributed to differences in the type of registry (national hospital-based vs. regional population-based) [22], the time period considered (perinatal vs. neonatal) [42], and the populations studied (all births vs. postmortems) [45]. Because 29.8% of CDH cases survived the perinatal period, we were unable to obtain information on subsequent treatment. However, other studies have confirmed that live births with CDH almost always receive treatment [24].

The rate of preterm delivery in our study appeared to be higher than that reported by another registry [7, 46]. CDH cases with a gestational age of less than 37 weeks were classified as premature, regardless of whether they resulted in live births, stillbirths, or terminations in our study. After excluding cases of pregnancy termination, the rate in our study was slightly lower than that reported in other studies [7, 46], although the difference was not statistically significant. As such, we hypothesized that the high rate of preterm birth observed in our study may be partly attributable to the high rate of pregnancy termination. In agreement with the findings of Shanmugam et al. [46], our study found that CDH cases with associated anomalies were more likely to result in premature delivery than isolated CDH cases. Similarly, when terminations were excluded from our analysis, a higher proportion of CDH cases with associated anomalies were born prematurely compared to isolated cases.

Infants with associated CDH were at a higher risk of perinatal death (80.1%) compared with those affected by isolated CDH (63.2%), consistent with previous reports [1, 22, 43]. In addition to cardiovascular system defects, there were more musculoskeletal diseases and neurological diseases among deceased patients, indicating that multiple malformations, especially circulatory, musculoskeletal, and central nervous system anomalies, are important factors in perinatal mortality.

Using 13 years of surveillance data and covering 24 million births, this study represents the most extensive investigation to date on the birth prevalence and time trend of CDH in the Chinese population. The wide geographical coverage, consistent case ascertainment methods, and adjustment for several characteristics ensure reliable estimates of the prevalence of CDH and the potential relationship between the maternal and infant characteristics and risk for CDH.

This study has several limitations. Firstly, the observed CDH prevalence may be underestimated due to hospital-based samples with a short monitoring period and incomplete population coverage. Secondly, information on chromosomal anomalies and syndromes for some CDH was unavailable due to limited chromosomal testing and syndromic case ascertainment in some member hospitals. Finally, CDH cases could not be classified based on the anatomical position of the defect, as the hernia types were not included in the routine data collection.

In conclusion, our study found that the Chinese population has a relatively low risk for CDH, but challenges remain. Cardiovascular anomalies are most commonly associated with CDH. The increasing trend in prevalence, demographic risk factors, and high perinatal mortality rate highlight the need for further research on the etiology, epidemiology, and clinical management of CDH in the contemporary Chinese population.

### Supplementary Information

Below is the link to the electronic supplementary material.

Supplementary file 1 (PDF 88 KB)

## Data Availability

The Chinese Birth Defects Monitoring Network database is not open access publicly available. The corresponding author obtained permission to use the data for this analysis from the National Health Commission of China. The datasets used and analyzed during the study are available from the corresponding author on reasonable request.
