# Supplementary material for: Epidemiology of congenital diaphragmatic hernia among 24 million Chinese births: a hospital-based surveillance study
Source: World J Pediatr. 2023 Dec 9;20(7):712–22. doi: 10.1007/s12519-023-00774-y (PMC11269495; doi:10.1007/s12519-023-00774-y)
Supplement: Supplementary file 2 — Supplementary file 1 (PDF 88 KB) [file 12519_2023_774_MOESM1_ESM.pdf]

Supplementary Table 1. Diagnosis time of congenital diaphragmatic hernia cases in Chinese newborns[a]

Diagnosis time*	Overall (*N* = 4396)	Isolated (*n* = 2737)	Associated (*n* = 1659)
Postpartum[b]	553 (12.6)	410 (15.0)	143 (8.6)
Antenatal	3843 (87.4)	2327 (85.0)	1516 (91.4)
Antenatal (wk)*			
< 28	2373 (61.7)	1313 (56.4)	1060 (69.9)
≥ 28	1470 (38.3)	1014 (43.6)	456 (30.1)

Data are presented as *n* (%). [a]One case with unknown diagnosis time were excluded; [b]within seven days after delivery. *Differed significantly between isolated and associated;

Supplementary Table 2. Comparison of characteristics between surviving and deceased congenital diaphragmatic hernia cases in Chinese newborns

Characteristics	Surviving[a] (*n* = 1306)	Deceased[b] (*n* = 3078)
Infant sex[c]*		
Male	789 (60.5)	1639 (55.1)
Female	515 (39.5)	1338 (44.9)
Maternal residence		
Urban	863 (66.1)	1932 (62.8)
Rural	443 (33.9)	1146 (37.2)
Maternal age*		
< 20	14 (1.1)	52 (1.7)
20-24	216 (16.5)	603 (19.6)
25-29	521 (39.9)	1287 (41.8)
30-34	360 (27.6)	734 (23.8)
≥ 35	195 (14.9)	402 (13.1)
Geographic region*		
Central	395 (30.2)	1077 (35.0)
East	649 (49.7)	1255 (40.8)
West	262 (20.1)	746 (24.2)
Diagnosis time[d]*		
Antenatal	910 (69.7)	2920 (94.9)
Postpartum	396 (30.3)	157 (5.1)
Gestational age (wk)[e]*		
< 37	231 (17.7)	2663 (86.6)
37-42	1064 (81.5)	399 (13.0)
≥ 42	11 (0.8)	15 (0.5)
Isolated or associated*		
Isolated	977 (74.8)	1753 (57.0)
Associated	329 (25.2)	1325 (43.0)

Data are presented as *n* (%). [a]Live within perinatal period; [b]including cases of stillbirths and neonate death within 7 days; [c]103 cases with unknown/unspecified gender were excluded; [d]one case with unknown diagnosis time were excluded; [e]two cases with unknown gestational age were excluded. *Differed significantly between survived and died

Supplementary Table 3. Comparison of associated abnormalities characteristic between surviving and deceased congenital diaphragmatic hernia (CDH) cases in Chinese newborns

Abnormalities associated with CDH*	Surviving[a] (*n* = 329)	Deceased[b] (*n* = 1325)
Nervous system	14 (4.3)	270 (20.4)
Eye, ear, face and neck	13 (4.0)	47 (3.6)
Circulatory system	213 (64.7)	783 (59.1)
Respiratory system	29 (8.8)	170 (12.8)
Cleft lip and cleft palate	19 (5.8)	116 (8.8)
Digestive system	25 (7.6)	69 (5.2)
Genital organs	25 (7.6)	25 (1.9)
Urinary system	17 (5.2)	146 (11.0)
Musculoskeletal system	40 (12.2)	268 (20.2)
Chromosomal abnormalities	6 (1.8)	45 (3.4)

Data are presented as *n* (%). [a]Live within perinatal period; [b]including cases of stillbirths and neonate death within 7 days. [*]Differed significantly between survived and died
